# Two Decades of Wildlife Pathogen Surveillance: Case Study of *Choclo orthohantavirus* and Its Wild Reservoir *Oligoryzomys costaricensis*

**DOI:** 10.3390/v15061390

**Published:** 2023-06-17

**Authors:** Publio Gonzalez, Jacqueline R. Salazar, Tybbysay P. Salinas, Mario Avila, Jocelyn P. Colella, Jonathan L. Dunnum, Gregory E. Glass, Gloria Gonzalez, Enos Juarez, Kimberly Lindblade, Edwin Pile, Yaxelis Mendoza, Juan Miguel Pascale, Anibal G. Armien, Joseph A. Cook, Blas Armien

**Affiliations:** 1 Department of Research in Emerging and Zoonotic Infectious Diseases, Gorgas Memorial Institute of Health Studies, Panama City 0816-02593, Panama; pgonzalez@gorgas.gob.pa (P.G.); jsalazar@gorgas.gob.pa (J.R.S.); tsalinas@gorgas.gob.pa (T.P.S.); ejuarez@gorgas.gob.pa (E.J.); pileedwin@gmail.com (E.P.); 2 Department of Vector Control-Herrera Health Region, Ministry of Health, Panama City 0843-03441, Panama; mario281159@yahoo.com; 3 Biodiversity Institute & Department of Ecology and Evolutionary Biology, University of Kansas, Lawrence, KS 66045, USA; colella@ku.edu; 4 Department of Biology and Museum of Southwestern Biology, University of New Mexico, Albuquerque, NM 87131, USA; jldunnum@unm.edu; 5 Department of Geography & Emerging Pathogens Institute, University of Florida, Gainesville, FL 32611, USA; gregglass1066@gmail.com; 6 Department of Genomics and Proteomics, Gorgas Memorial Institute of Health Studies, Panama City 0816-02593, Panama; ggonzalez@gorgas.gob.pa (G.G.); ygmendoza@gorgas.gob.pa (Y.M.); jmpascale@gorgas.gob.pa (J.M.P.); 7 Malaria Branch, Division of Parasitic Diseases and Malaria, Centers for Disease Control and Prevention, Atlanta, GA 30329, USA; kalindblade@gmail.com; 8 Sistema Nacional de Investigación (SNI), Secretaria Nacional de Ciencia y Tecnología (SENACYT), Panama City 0816-02852, Panama; 9 California Animal Health & Food Safety Laboratory System (CAHFS), School of Veterinary Medicine, University of California, Davis, CA 95616, USA; agarmien@ucdavis.edu

**Keywords:** *Oligoryzomys*, One Health, *Orthohantavirus*, spatial ecology, wildlife surveillance, zoonotic disease

## Abstract

The Costa Rican pygmy rice rat (*Oligoryzomys costaricensis*) is the primary reservoir of *Choclo orthohantavirus* (CHOV), the causal agent of hantavirus disease, pulmonary syndrome, and fever in humans in Panama. Since the emergence of CHOV in early 2000, we have systematically sampled and archived rodents from >150 sites across Panama to establish a baseline understanding of the host and virus, producing a permanent archive of holistic specimens that we are now probing in greater detail. We summarize these collections and explore preliminary habitat/virus associations to guide future wildlife surveillance and public health efforts related to CHOV and other zoonotic pathogens. Host sequences of the mitochondrial cytochrome *b* gene form a single monophyletic clade in Panama, despite wide distribution across Panama. Seropositive samples were concentrated in the central region of western Panama, consistent with the ecology of this agricultural commensal and the higher incidence of CHOV in humans in that region. Hantavirus seroprevalence in the pygmy rice rat was >15% overall, with the highest prevalence in agricultural areas (21%) and the lowest prevalence in shrublands (11%). Host–pathogen distribution, transmission dynamics, genomic evolution, and habitat affinities can be derived from the preserved samples, which include frozen tissues, and now provide a foundation for expanded investigations of orthohantaviruses in Panama.

## 1. Introduction

Hantaviruses (Order *Bunyavirales*, Family *Hantaviridae*) comprise a group of negative-sense RNA viruses that are distributed worldwide. While rodents and eulipotyphlans (shrews and moles) have been advanced as the primary hosts of hantaviruses globally, new strains have recently been detected in chiropterans [1], reptiles, and fish [2]. As a result, four subfamilies are recognized within Hantaviridae: Mammantavirinae (*Loanvirus*, *Mobatvirus*, *Orthohantavirus*, and *Thottimvirus*), the bat-, mole-, shrew-, and rodent-borne viruses; Repantavirinae (*Reptillovirus*), the reptile-borne viruses; Actantavirinae (*Actinovirus*), the ray-finned fish-borne viruses; and Agantavirinae (*Agnathovirus*), the jawless fish-borne viruses [3]. In general, hantaviruses trend towards host specificity (one virus, one host [4]); however, there have been instances of multiple strains detected in a single host or the same strain found in multiple different host species [5,6,7,8]. To date, only rodent-borne orthohantaviruses have been associated with human disease.

Zoonotic infection of humans by hantaviruses occurs through the inhalation of aerosols contaminated by the urine or feces of an infected host [9]. Some New World hantaviruses cause hantavirus pulmonary syndrome (HPS) or hantavirus cardiopulmonary syndrome (HCPS), which can lead to fatal cardiac shock in humans [10,11], whereas Old World hantaviruses cause hemorrhagic fever with renal syndrome (HFRS). In the Americas, HCPS is characterized as a “flu-like illness” with gastrointestinal symptoms that can range from mild to severe. Orthohantavirus diseases have a mortality rate ranging from 12% (HFRS) to 40% (HCPS) [12], although many milder cases may go unreported [13]. In contrast, rodent hosts appear to be relatively unaffected [14], making them both reservoirs and zoonotic vectors. Establishment of laboratory animal models for hantavirus is challenging [15,16] due to the risks to personnel, which necessitates expanded investigation of wild hosts and positions hantaviruses as a model for a One Health (human–animal–environment) approach towards emerging infectious diseases.

In the early 2000s, an acute outbreak of HCPS on the Azuero Peninsula in the province of Los Santos in central Panama led to the first documentation of hantavirus in the country [17,18]. The causal agent, *Orthohantavirus chocloense* [19], has since been isolated and sequenced from both humans and its primary wildlife reservoir, *Oligoryzomys fulvescens* (=*costaricensis*) [20,21], the Costa Rican pygmy rice rat [22,23]. Previous ecological studies of CHOV and *Oligoryzomys*, along with human epidemiological surveillance, have characterized a “CHOV-endemic region” in central-western Panama [18,24,25,26,27]. Yet until recent years, the eastern and northern regions of the country had been undersampled and understudied, so it was unclear whether CHOV was not present there or simply undetected. Understanding where the host and virus are distributed across the landscape is a necessary first step in the identification of risk areas for emergence that forms future public health guidance and future surveillance efforts of both wildlife and people.

Understanding how viral prevalence changes over time is critical and hinges on the availability of temporally deep wildlife archives. In the case of CHOV, the wild host is known to experience seasonal population cycles [28] that may also affect the prevalence and distribution of the disease [29]. In areas unaltered by agriculture, both the spatial distribution and abundance of *O. costaricensis* vary seasonally and inter-annually, with abundance generally lowest in December during the transition from wet to dry season and populations reestablishing and then increasing in abundance during the dry season [28]. Such demographic fluctuations are less prominent in agriculturally modified areas where host reproductive output can occur year-round unrestricted by the availability of food [30] This pattern suggests that agricultural development may have driven CHOV emergence in Panama [5,22,31]. Increased abundance of *O. costaricensis* in response to excess agricultural food resources may increase host density, elevate pathogen prevalence, and ultimately increase risk of zoonotic spillover and human infection [22,26,28,32,33]. Agricultural development also reduces biodiversity, which can unintentionally lead to higher prevalence of a disease in remaining host species [34]. To this end, it is important to understand associations between host distribution and abundance, viral prevalence, and environments across both space and time. 

We conducted holistic mammal surveys from 2000 to 2019. We obtained mitochondrial barcodes and seroprevalence data from the rodent hosts to document the spatial extent of *O. costaricensis* and CHOV. We explored host distribution and geographic variation in Panama and assessed the viral presence across five microhabitat categories (cropland, pasture, secondary vegetation, shrub, and peridomestic [26]) within five of the seven ecoregions in Panama to better understand CHOV prevalence across space and time. 

## 2. Materials and Methods

### 2.1. Study Area and Small Mammal Surveys

A survey of non-volant small mammals in Panama was undertaken from February 2000 to December 2019. Panama supports diverse habitats with an elevational range from sea level to 3475 m, annual average precipitation varies from 1200 to 7000 mm, and temperatures span 7–27 °C. The total terrestrial area of the country is 74,177 km^2^ and approximately 40% of that is agricultural or pastureland [35,36]. Generally, Panama is hot and humid along the coasts, while the interior experiences greater environmental variation dependent on elevation [37]. 

Survey methods followed animal care and use procedures as outlined by the American Society of Mammalogists [38,39]. Specimens were holistically collected [40], morphologically identified to species level, and collaboratively preserved at the Museum of Southwestern Biology at the University of New Mexico, the Vertebrate Museum of the University of Panama, or the Zoological Collection of the Gorgas Memorial Institute for Health Studies to maximize the utility of the collected samples to the extended biodiversity and public health communities. Standard measurements, sex, reproductive status, and GPS coordinates associated with the collection locality (WGS 1984) were recorded at the time of collection for all animals [41]. These data are publicly available through the Arctos database (https://arctosdb.org, accessed on 30 April 2023). To maximize consistency, one investigator recorded 80% of the measurements over the 19 years of sampling (MA). We emphasize the value of long-term collection efforts in enabling temporally deep and geographically broad public health perspectives on emerging zoonotic diseases [42].

### 2.2. Sequencing and Serology

**Figure 1:**
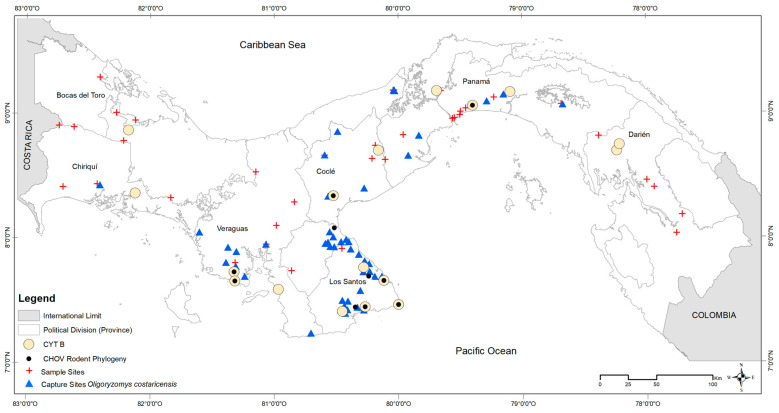
Mammal capture site distribution in Panama, 2000–2019. Total mammal capture sites (red crosses), *Oligoryzomys costaricensis* capture sites (blue triangles), sites where CHOV was amplified in *Oligoryzomys costaricensis* (black dots), and sites chosen for the identification of *Oligoryzomys costaricensis* by *cytb* (cream circles) are represented.

To verify morphological host species determinations and assess the potential geographic variation of *O. costaricensis* within Panama, we sequenced part of the mitochondrial cytochrome b (*cytb*, 1140 bp) gene as a molecular barcode for host identification. Representatives from populations spanning its Panamanian distribution were selected for molecular analysis. DNA was extracted from the spleen, liver, or kidney from 33 host specimens (Supplementary Table S1) using a QIAamp DNA Mini Kit (Qiagen Inc., Valencia, CA, USA) following the manufacturer’s protocols. We included at least two individuals from each of seven provinces of Panama: Cañazas, Bocas del Toro; Boca del Monte, Chiriquí; Aguas Claras, Colón; Tamarindo and Zimba, Darién; El Bebedero, Cañas, Barriada 8 de Noviembre, San José, Pocrí, and Punta Mala, Los Santos; Santa Rosa Abajo and Tocumen, Panamá; Malena, La Zumbona, and Punta San Lorenzo, Veraguas (see Figure 1). 

Partial *cytb* was PCR-amplified for each host using primers MVZ05 (5′- CGAAGCTTGATATGAAAAACCATCGTTG—3′ [43]) and MVZ14 (5′—GGTCTTCATCTYHGGYTTACAAGA—3′ [44]). PCR reactions were performed using Taq PCR Master Mix (Qiagen Inc.) with 1.5 mM MgCl_2_, 0.2 mM dNTPs, 0.4 ρmol of forward and reverse primers, 2.5 units of Taq polymerase, and 1 μL template DNA (50 ng/μL) for a final volume of 25 μL with the following thermal cycling conditions: 94 °C for 3 min, 30 cycles at 94 °C for 30 s, 45 °C for 90 s, 72 °C for 90 s, and a final extension for 10 min at 72 °C. Products were visualized on 1.5% agarose gel. Amplicons were cleaned using the QIAquick PCR Purification Kit (Qiagen). Sequencing reactions were performed using the ABI BigDye Terminator v. 3.1 Cycle Sequencing Kit (Thermo Fisher Scientific, Waltham, MA, USA) with an ABI PRISM 3130xl Genetic Analyzer (Life Technologies, Carlsbad, CA, USA). All sequences were assembled and aligned using Sequencher v. 4.6 (GeneCodes, Ann Arbor, MI, USA). 

**Figure 2:**
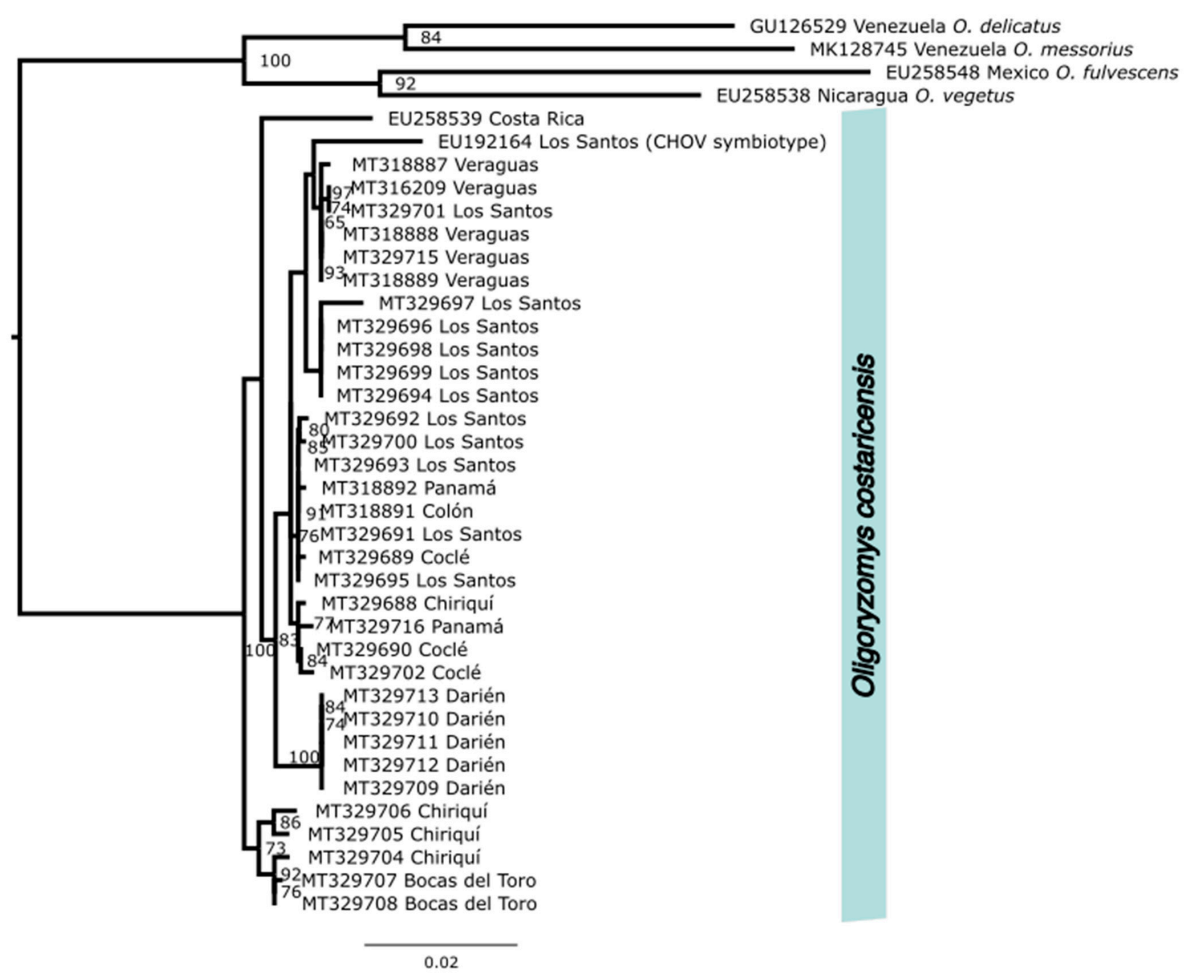
Maximum likelihood tree of mitochondrial cytochrome b sequences for 34 Panamanian and 1 Costa Rican *Oligoryzomys costaricensis* and four outgroups.

We used NCBI’s (National Center for Biotechnology Information) Basic Local Alignment Search Tool (BLAST [45]) to identify two additional *cytb* sequences from *O. costaricensis* (EU192164, EU258539), which were available on GenBank. Those 35 sequences were combined with *cytb* sequences from four outgroup species also from the Neotropics, *O. fulvescens* (EU258548), *O. vegetus* (EU258538), *O. delicatus* (GU126529), and *O. messorius* (MK128745), which were used to root the phylogeny. Sequences were aligned using MUSCLE [46] and maximum likelihood phylogeny was inferred using IQ-TREE, V.2.2.2.6 (Nguyen et al. 2018) with automated model selection, which was conducted through ModelFinder [47]. Trees were generated with 1000 ultrafast bootstrap alignments repeated a maximum of 1000 times, and the consensus species tree was visualized using Fig Tree v 4.2 (http://tree.bio.ed.ac.uk/software/figtree/) with text adjustments in InkScape (InkScape Project 2020; inkscape.org/, accessed on 10 May 2023) (see Figure 2). 

**Figure 3:**
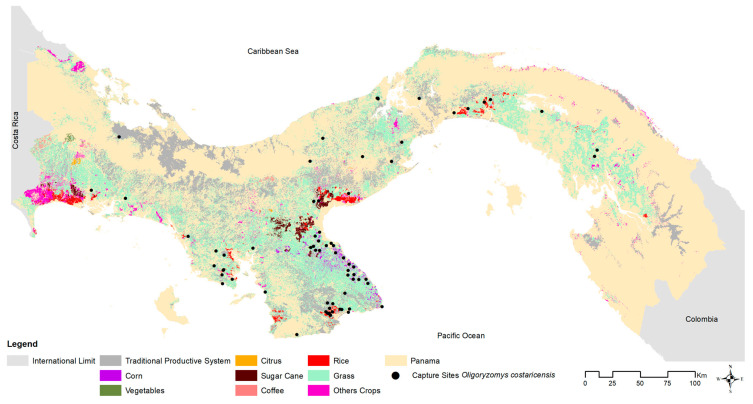
*Oligoryzomys costaricensis* capture site distribution and land use cover in Panama, 2000–2019. The black dots represent the capture sites of *Oligoryzomys costaricensis*. Common agricultural types are represented by color palette.

**Figure 4:**
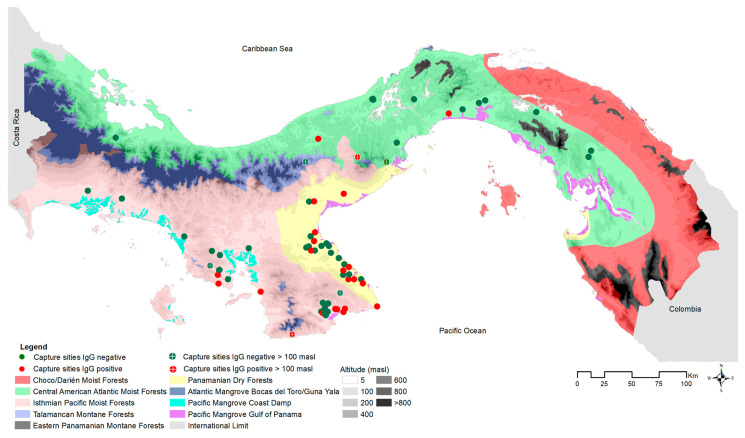
*Oligoryzomys costaricensis* capture site distribution by ecoregion and altitude limit in Panama, 2000–2019. Each dot represents a *Oligoryzomys costaricensis* capture site: IgG positive at <100 masl (meters above sea level; red dots), IgG negative at <100 masl (green dots), IgG positive at >100 masl (red dots with white cross), and IgG negative at >100 masl (green dots with white cross). The color palette shows the nine ecoregions, and the elevation is shown using a gray gradient from 5 to >800 masl.

To test for current or prior hantavirus infection in the host, blood samples from 778 of the 883 captured *O. costaricensis* were screened for antibodies using an IgG strip immunoblot assay [48]. Immature individuals (<10 g) were removed from analysis to avoid inflating prevalence estimates with potential transovarial transmission [5,49,50], leaving 626 adults. Positive and negative results were visualized in geographic space using ArcGIS v. 10.7 (ESRI 2019), compared against microhabitat (cropland, peridomestic [e.g., households, adjacent outbuildings, gardens, livestock enclosures], pasture, shrub, and secondary vegetation), and ecoregion (see Figure 3 and Figure 4). Positive cases were summed by microhabitat category within each ecoregion [26]. 

### 2.3. Environmental Associations

About 44% of Panama is wooded, 39% is rural agricultural (crops and pasture, [26,35], and 2% is multi-use, which includes towns and industrial centers [35,51]. To explore the association between host, viral presence, and general environmental composition, each site was classified at two coarse spatial scales: (1) microhabitat, (cropland, pasture, peridomestic [e.g., households, adjacent outbuildings, gardens, livestock enclosures], secondary vegetation, and shrub), and (2) ecoregion [51]. There are 9 distinct ecoregions (7 terrestrial, 2 marine) in Panama. We sampled one coastal ecoregion (Pacific Mangrove South America) and six other terrestrial ecoregions: (1) Central American Atlantic Moist Forests, which includes Ngäbe-Buglé, one of Panama’s five ‘comarcas indígenas’ formerly belonging to the provinces of Bocas del Toro, Chiriquí, and Veraguas; (2) Talamancan Montane Forests; (3) Isthmian-Pacific Moist Forests; (4) Panamanian Dry Forests; (5) Choco/Darién Moist Forests; and (6) Eastern Panamanian Montane Forest (see Supplementary Table S2). Within the last 80 years [52], much of the historical Central American Atlantic Moist Forests, Isthmian-Pacific Moist Forests, and Panamanian Dry Forest ecoregions have been converted to agricultural lands (67% cattle pasture and 5% crops [rice, corn, and cane sugar]). To account for sampling biases across ecoregions, the total number of captures in each environmental type (microhabitat categories, ecoregion) was scaled by the total number captured in that environment.

### 2.4. Statistical Analyses

Continuous and categorical variables were analyzed using EPIINFO Version 7.2.4.0 (Centers for Disease Control and Prevention, Atlanta, GA, USA) and assessed using parametric and nonparametric techniques. A *p*-value with alpha <0.05 was considered significant.

## 3. Results

### 3.1. Biorepository Development & Spatial Distributions of Host & Pathogen

This 20-year surveillance project generated >10,500 specimens representing 110 species of non-volant small mammals distributed throughout Panama. This biodiversity archive provided critical biological material for the two recognized hosts of two orthohantaviruses in Panama, *O. costaricensis* (CHOV) and *Zygodontomys brevicauda*, the primary host of Calabazo virus [17]. The archive also built comprehensive sampling of the associated mammalian communities for diverse other studies. All specimens were holistically prepared, including heart, lung, kidney, spleen, and blood samples, cryogenically preserved, and archived at the Gorgas Memorial Institute, the Vertebrate Museum of the University of Panama, and Museum of Southwestern Biology (MSB) in Albuquerque, New Mexico. Traditional host voucher specimens were archived in the MSB Division of Mammals. Tissues (heart, liver, kidney, spleen) were cryogenically preserved in nitrogen in the field and permanently archived in ultracold freezers (−80 °C) in the Gorgas Memorial Institute or in nitrogen vapor tanks (−190 °C) in the MSB Division of Genomic Resources. Data associated with each specimen are openly available through the Arctos museum database and physical specimens can be loaned from the institutions.

In total, 833 wild *O. costaricensis* were collected from 2000 to 2019. Of those, 380 specimens collected between 2000 and 2006 formed the basis for earlier investigations [22,24,26,33]. Here, we add an additional 453 samples to extend the geographic extent and temporal span of earlier samples to include 2007 to 2019 (see Figure 1). Collection localities were widespread, reaching from the southwestern border of Panama and Costa Rica to 40 km east of the Panama Canal. Of the 155 sites sampled for rodents, *O. costaricensis* were detected at 71 sites. Occurrences were consistent with the previously described distribution of *O. costaricensis* in Panama [53], but we document important new records in Ngäbe-Buglé to the north and also eastward into Darién (see Supplementary Figure S1), potentially related to agricultural development and host range expansion. About 93% of *O. costaricensis* captures occurred below 100 m elevation, with only six exceptions, each a single capture event: Veraguas (El Jagua, Cerro Hoya), Coclé (El Cope National Park, San Miguel Centro), Panama (Altos de Campana National Park), and Los Santos (Oria) (see Figure 4). The highest elevational record of *O. costaricensis* was at 797 m.

Costa Rican pygmy rice rats were frequently detected in anthropogenically transformed rural areas (91%; 65 of 71 sites) or highly-disturbed urban areas (3%; 2 sites), as opposed to intact natural areas (6%; 4 sites). Among ecoregions (see Figure 1), 42% of all sites where *O. costaricensis* were recorded were in the Isthmian-Pacific Moist Forest (30 of 71 sites) and 34% were in the Panamanian Dry Forest (24 of 71 sites there). There was only one record each from the Talamancan Montane Forest and Pacific Mangrove South America ecoregions. Although there were four sampling sites in Choco/Darién, no *O. costaricensis* were detected there. Only 21% of all sites in Panama where *O. costaricensis* were detected were located in the Central American Atlantic Moist Forest ecoregion (15 of 71 sites), the largest ecoregion in Panama. Two sites (Zimba, Tamarindo) in this ecoregion located in Darién province had *O. costaricensis*, representing the most eastern records for the species. These extend the known distribution of *O. costaricensis* [53] by ca. 120 km to the east (see Supplementary Figure S1). Finally, for the total number of individual captures of *O. costaricensis* across microhabitat categories, 28% (234) of *O. costaricensis* captures occurred in croplands, 26% (217) in peridomestic sites, and 39% (322) in pastures.

### 3.2. Sequencing and Serology

We generated high-quality sequence data from host tissues cryogenically preserved in museum collections. Partial *cytb* sequences from *O. costaricensis* formed a single monophyletic group with 100% bootstrap support, consistent with morphological species diagnoses that a single species of pygmy rice rat occurs in Panama (see Figure 2) with generally minimal substructure. Within the Panamanian clade, however, potential geographic substructure was identified with a well-supported Darien clade. Our species records extend the documented range of pygmy rice rats east into Darién (see Supplementary Figure S1) and north into Ngäbe-Buglé. Preliminary mitochondrial sequence data identified pygmy rice rats from Costa Rica as ancestral to those in Panama. Two major mitochondrial clades were detected within Panama, one distributed in the eastern provinces of Chiriquí and Bocas del Toro and another distributed throughout the rest of Panama. 

Hantavirus seroprevalence in *O. costaricensis* was 16% (122/778) overall. After removing immature individuals, adult seroprevalence levels increased to 18% (111/626), with significant differences between male (22.5% [87/386]; 95% CI = 19.0, 27.0) and female (10.00% [24/240]; 95% CI = 7.0–15.0%) prevalence across sites (X^2^ = 15.10; *p* = 0.0001). Seroprevalence data were summarized by ecoregion and microhabitat (see Table 1). 

The highest prevalence coincided with the historically recognized endemic area of the hantavirus disease [24,26,54], but 3 of 15 *O. costaricensis* individuals sampled outside the area of endemism tested positive.

## 4. Discussion

We summarize two decades of hantavirus field studies in Panama that were initiated following the emergence of CHOV in late 1999. We update the known distributional limits of *O. costaricensis*, the primary host of CHOV, and demonstrate limited molecular variation across Panama, with the exception of Darien specimens, based on a single mitochondrial DNA barcode. Additional independent nuclear markers and more comprehensive geographic sampling of the host in Panama, Costa Rica, and Colombia, with a focus on undisturbed areas, should now be developed. Such molecular investigations could explore in more detail potential geographic variations in the host, demographic and phylodynamic history (e.g., expansion and contraction), and biogeographic origins. 

We then associated the geographic distribution and relative abundance of *O. costaricensis* in Panama with the prevalence of CHOV antibody detection across multiple ecoregions. Human CHOV cases, reported outside of this investigation, primarily occur in agricultural communities in the central region of Panama (Los Santos, Herrera, Coclé, and Veraguas provinces [55]) likely due to local abundance of granivorous rodents in response to excess food availability. A greater abundance of rodents increases the opportunity for contact with and zoonotic transmission to humans, especially for those working and living close to agricultural fields. Through our long-term screening efforts, we have extended the understanding of the Costa Rican pygmy rice rat as the primary wild reservoir of CHOV in Panama. Consistent with previous investigations, we document a wide geographic distribution of the host [18,22,24,26,31,53], with pygmy rice rats found in four of seven terrestrial and coastal ecoregions. Seropositive hosts were identified across four ecoregions and five distinct microhabitats. All habitats where *O. costaricensis* were detected have been modified somewhat by human perturbation (e.g., cropland, pasture, shrub, secondary vegetation, and peridomestic). New host records in the Central American Atlantic Moist Forests (Ngäbe-Buglé) and Eastern Panamanian Montane Forest (Darién) ecoregions suggest that the host species may have expanded northward and eastward coincident with regional agricultural expansion [56,57]; however, the species may have simply been undetected until now. 

A pathogen always has a broader spatial distribution than that of the disease itself [58] and a narrower distribution than that of its hosts. Consistent with this hypothesis, we did not record CHOV-positive mice throughout the entire geographic range of *O. costaricensis*. Instead, we found higher prevalence of CHOV in disturbed rural areas compared to natural areas, although our sampling was primarily focused on the former. Agricultural proximity is associated with sustained, year-round reproduction in *Oligoryzomys*, presumably because such areas provide an abundance of supplementary and stable food resources [30]. We hypothesize that higher host densities translate to higher pathogen densities. Support for this scenario has been documented for other rodent–hantavirus systems in South America, where host population density and prevalence of hantavirus infection were significantly higher in peridomestic habitats [59]. 

Understanding the natural history of the host, in addition to the distribution of the pathogen, is essential to refining our understanding of the spatial distribution of the disease and forecasting how that distribution might change in the future. In the case of orthohantaviruses in Panama, expanded agricultural development may have facilitated an expanded geographic distribution of the host (e.g., captures west of the Panama Canal, where previous surveys from 1970–1977 had not detected the species) and also led to increased host population sizes through elevated reproductive output. These possibilities should be further monitored and rigorously tested. Previous longitudinal studies of small mammals and *Sin Nombre orthohantavirus* in the southwestern United States [41] demonstrated a positive relationship between increased resource availability due to natural environmental change (El Niño Southern Oscillation [ENSO] events), rodent density, and subsequent changes in hantavirus prevalence. Human-induced habitat changes have the potential to artificially mirror naturally occurring events, particularly when the reservoir species involved are well-adapted for rapid response to favorable conditions (e.g., cricetid rodents). Hosts for zoonotic viruses are more likely to be opportunistic, generalist species that frequently inhabit anthropogenically disturbed habitats [60]. Wildlife surveillance that targets agricultural interfaces near human city centers may be most effective at detecting and subsequently mitigating regional hantavirus outbreaks. Further, forest restoration was shown to decrease the abundance of hantavirus reservoir rodents, including *Oligoryzomys* (from 89% to 43%), thereby decreasing the chance of zoonotic transmission by ~45% [57]. Expanded sampling and regular resurveys across representative environments remains critical, especially as anthropogenic impact on the environment induces change in natural communities. Substantial environmental heterogeneity in western Panama is associated with elevated endemism in rodents, which may also contribute to patterns of zoonotic transmission, but sampling remains too limited to effectively explore this possibility.

This work was limited by several assumptions that require further exploration. First, many pathogens, including hantaviruses [61], are capable of infecting multiple host species [8] but being able to infect a host is not the same as being productively infectious. Second, seroprevalence is not a measure of active infection and, although serological data for hosts <10 g were excluded from analysis, the possibility and frequency of transovarial transmission of the virus or maternal antibodies remain unknown. That knowledge gap challenges our ability to extend these test results into environmentally and spatially explicit models [5,50]. We only generated sequence data for a subset of seropositive hosts and only from a single mitochondrial marker; therefore, additional genetic structure within the host may be yet undetected. More comprehensive assessment of host genomic variation might provide key insight into the host colonization dynamics of newly disturbed habitats in Panama. Host phylogeography also can provide insights into mechanisms underlying viral evolution [62]. Given that these specimen collections were accumulated over the last two decades, insight into temporal aspects of both host neutral and immune-related genes [63] as well as *Orthohantavirus* evolution can now be pursued. Full genome sequencing of the hosts and viruses should be a priority as we aim to understand issues such as mutation rates, reassortment, and potential interactions with other co-circulating orthohantaviruses in these mammalian communities.

## 5. Future Directions

The Gorgas Memorial Institute’s approach to CHOV field studies in Panama emphasizes the development of temporally deep and spatially broad biorepositories of both host and pathogen samples. Now, after 20 years of site-intensive sampling, questions related to CHOV evolution and ecology are tractable, reinforcing the value of holistic collecting [40] of wildlife hosts and their associated parasites, endoparasites, and pathogens. The One Health approach is predicated on building long-term biorepositories to allow for the replication and extension of studies and as technology improves, these historic samples allow us to more fully understand host–pathogen dynamics in addition to the distribution and ecology of emerging zoonotic diseases [64,65,66]. 

Although not explored in detail here, preliminary sampling in undisturbed natural ecosystems (e.g., Parque Nacional Amistad, Vulcan Baru) has identified other potential seropositive hosts for orthohantavirus not included here, such as *Peromyscus nudipes*, *Reithrodontomys mexicanus*, *R. sumichrasti*, *R. creper*, *Sigmodon hirsutus*, *Liomys adspersus*, and *Transandinomys talamancae*, which we are now characterizing. An incomplete understanding of the mammalian communities that may serve as reservoirs for CHOV or other yet unrecognized orthohantaviruses in Panama may complicate interpretations of the spatial distribution of the pathogen and risk landscape. Other orthohantaviruses, such as Calabazo virus that has been found primarily in *Z. brevicauda*, are circulating in these mammalian communities but have been only minimally surveyed [17,22]. Survey efforts will require viral screening of specimens of other wild mammalian hosts that are now available through these archival collections built over two decades of fieldwork. Understanding potential interactions among the diverse pathogens circulating in these communities may be facilitated by the application of new metatranscriptomic or metagenomic sequencing methods [67]. More extensive and detailed genetic characterization and analyses of both host and virus are needed to test these preliminary findings using phylodynamic methods and disease modeling, among other approaches [68,69]. Such analyses will help to characterize the virus’ evolution and identify the suite of environmental and ecological conditions suitable for the host and the subset of conditions in which the virus is found.

While we use gross correlative methods to characterize broad environmental associations among hosts, pathogens, and their environment, the next step will be to incorporate these data into an ecological niche modeling framework [70]. Such a framework will allow us to visualize how the suitability of the landscape for each interacting component changes across space and time and to identify potentially causal features useful for building predictive models of emerging disease. Habitat conversion, for example, is now opening the Darién Gap to expansion by commensal mammalian species likely to cause disease outbreaks (e.g., grassland species such as *Oligoryzomys*, livestock-fed *Desmodus* vampire bats, and edge-exploiting species such as *Didelphis* marsupials). Pairing this habitat conversion with increasing human migration through the Panamanian Isthmus suggests the importance of wildlife pathogen surveillance to public health throughout the Americas.

## Figures and Tables

**Table 1:** Total captures of *Oligoryzomys costaricensis* and percent prevalence of IgG antibody against N protein by microhabitat * across five ecoregions in Panama. No seropositives were found in Choco/Darién Moist Forests, Eastern Panamanian Montane Forest, and Talamancan Montane Forest ecoregions.

Ecoregion	Total % (IgG+/n)
Central American Atlantic Moist Forests	8 (8/102)
Peridomestic *	13 (2/15)
Crops	4 (1/26)
Pasture	10 (5/56)
Shrubs	0 (0/2)
Secondary vegetation	0 (0/3)
Isthmian-Pacific Moist Forests	18 (66/374)
Peridomestic	16 (10/63)
Crops	25 (32/126)
Pasture	13 (21/163)
Shrubs	0 (0/1)
Secondary vegetation	14 (3/21)
Panamanian Dry Forests	16 (47/293)
Peridomestic	14 (15/106)
Crops	20 (14/71)
Pasture	15 (14/91)
Shrubs	14 (3/22)
Secondary vegetation	33 (1/3)
Pacific Mangrove S. America	13 (1/8)
Peridomestic	-
Crops	-
Pasture	25 (1/4)
Shrubs	0 (0/3)
Secondary vegetation	0 (0/1)
Total	16 (122/778)

* Disturbed environments are peridomestic, crops, pasture, shrubs, and secondary vegetation [26].

## Data Availability

All sequence data are available through NCBI’s GenBank (Accession numbers for *cytb* sequences: MT316209, MT318887, MT318892, MT329688, MT329716). Specimens and tissues are available through the Museum of Southwestern Biology at the University of New Mexico, the Vertebrate Museum of the University of Panama, or the Zoological Collection of the Gorgas Memorial Institute for Health Studies in Panama City, with digital data available through the Arctos database (arctosdb.org, accessed on 30 April 2023).

## Supplementary Materials

The following supporting information can be downloaded at: https://www.mdpi.com/article/10.3390/v15061390/s1, Figure S1: Historical and current spatial distribution of *O. costaricensis*. The map is created from the collection sites of museum specimens (striped lines) using as reference the map generated by Méndez (1993) [53]. The Hantavirus Project 2000–2019 distribution map creates a spatial model generated by circular polygons around capture points, assuming a dispersion range of 20 km; Table S1 ([43,44,71,72]): List of primers used for amplifications and sequencing of *Choclo orthohantavirus* S segment and *cytb* gene of *Oligoryzomys costaricensis*. Table S2: Distribution of ecoregions of Panama according to land use [35], area, and altitude.
