# Supplementary material for: Two Decades of Wildlife Pathogen Surveillance: Case Study of Choclo orthohantavirus and Its Wild Reservoir Oligoryzomys costaricensis
Source: Viruses. 2023 Jun 17;15(6):1390. doi: 10.3390/v15061390 (PMC10303383; doi:10.3390/v15061390)
Supplement: Supplementary file 1 [file viruses-15-01390-s001.zip › Supplementary Table S1. List of primers_12062023.pdf]

Supplementary Table S1. List of primers used for amplifications and sequencing of *Choclo orthohantavirus* S segment and Cyt*b* gene of *Oligoryzomys costaricensis*.

**Name**	**Sequence**	**Design by:**	**Purpose**
	**Cytochrome B gene**		
MVZ05	5'-CGA AGC TTG ATA TGA AAA ACC ATC GTT G-3'	Irwin et al. 1991	PCR, sequencing
MVZ14	5'-GGT CTT CAT CTY HGG YTT ACA AGA C-3'	Smith and Patton, 1993	PCR, Sequencing
MVZ16	5'-AAATAGGAARTATCAYTCTGGTTTRAT-3'	Smith and Patton, 1993	Sequencing
H15149	5'-AAACTGCAGCCCCTCAGAATGATATTTGTCCTCA-3'	Kocher et al. 1989	Sequencing
L14734	5'-AAA AAC CAT CGT CCT TTA TAG G-3'	Irwin et al. 1991	Sequencing
L15513	5'-CTA GGA GAC CCT GAC AAT CTA-3'	Irwin et al. 1991	Sequencing
UNFOR403	5'-TGA GGA CAA ATA TCA TTC TGA GG-3'	Kent and Norris, 2005	Sequencing
UNREV1025	5'-GGT TGT CCT CCA ATT CAT GTT A-3'	Kent and Norris, 2005	Sequencing

Irwin, D M et al. "Evolution of the cytochrome b gene of mammals." *Journal of molecular evolution* vol. 32,2 (1991): 128-44. doi:10.1007/BF02515385

Smith, M F, and J L Patton. "Variation in mitochondrial cytochrome b sequence in natural populations of South American akodontine rodents (Muridae: Sigmodontinae)." *Molecular biology and evolution* vol. 8,1 (1991): 85-103. doi:10.1093/oxfordjournals.molbev.a040638

Kocher, T D et al. "Dynamics of mitochondrial DNA evolution in animals: amplification and sequencing with conserved primers." *Proceedings of the National Academy of Sciences of the United States of America* vol. 86,16 (1989): 6196-200. doi:10.1073/pnas.86.16.6196

Kent, Rebekah J, and Douglas E Norris. “Identification of mammalian blood meals in mosquitoes by a multiplexed polymerase chain reaction targeting cytochrome B.” *The American journal of tropical medicine and hygiene* vol. 73,2 (2005): 336-42.
