# Supplementary material for: Two Decades of Wildlife Pathogen Surveillance: Case Study of Choclo orthohantavirus and Its Wild Reservoir Oligoryzomys costaricensis
Source: Viruses. 2023 Jun 17;15(6):1390. doi: 10.3390/v15061390 (PMC10303383; doi:10.3390/v15061390)
Supplement: Supplementary file 1 [file viruses-15-01390-s001.zip › Supplementary Table S2. Distribution of the ecoregions jac-10052023.pdf]

Supplementary Table S2. Distribution of the ecoregions of Panama according to land use (MiAmbiente, 2017), area and altitude.

Ecoregion	Region number	Description	Total area (km^2)	Panama	
				Area (km^2)	Altitude (masl)
1. Central American Atlantic moist forests-Guatemala, Belize, Honduras, Nicaragua, Costa Rica, Panama	13	Banana plantation and cattle ranch expansion, logging, clearing, and refugee settlements in Nicaragua all pose severe threats to the ecoregion. Exploitation of parrots and other wildlife is a further threat. Conversion and degradation, even in designated protected areas, will likely increase in the next decade.	155,020	23,476	0 - 1400
2. Talamancan montane forests-Costa Rica, Panama	16	The Talamancan montane forests are notable for their rich biotas and high number of regional and local endemic species. Burning, logging, and other conversion leading to intensive agricultural use are the major threats to these forests.	15,991	7,232	5- 3200
3. Isthmian-Pacific moist forests-Costa Rica, Panama	15	Logging, mining, burning, and habitat conversion for pasture and agriculture represent extreme and high-intensity threats to this ecoregion. Wildlife exploitation and pollution are additional threats.	28,881	19,598	0 - 2400
4. Panamanian dry forests-Panama	73	The ecoregion is threatened by grazing, burning, and exploitative hunting.	5,199.74	5,200	0 - 400
5. Choco/Darién moist forests-Colombia, Panama, Ecuador	39	The Choco/Darien ecoregion is considered to have one of the world's richest lowland biotas, with exceptional richness and endemism in a wide range of taxa including plants, birds, reptiles and amphibians, and butterflies. Unplanned colonization	82,079	13,266	0 - 1600

		following the completion of roads and massive logging concessions are major threats to the Choco forests. Since 1960, over 40 percent of the forests have been cleared or heavily degraded and deforestation rates are accelerating (Salaman, 1994). This ecoregion faces serious threats in the next five to ten years from national-level development projects, including dams, roads, seaports, pipelines, and military installations. Currently, intensive logging, human settlement, mining, wildlife exploitation, and coca cultivation all threaten the ecoregion.			
6. Eastern Panamanian montane forests- Panama, Colombia	40	The higher peaks of the Serrania de San Blas, Darien, Maje, and Pirre of central and eastern Panama are covered in tropical cloud forest. Both the flora and fauna of these relatively isolated ranges contain numerous endemic species and represent an unusual assemblage of species with South American and Central American affinities. Habitat and environmental quality are being degraded by mining, and numerous wildlife species are being exploited by overhunting for subsistence and trade.	2,905	2,654	5 - 1600
Atlantic Mangrove Complexes and Mangrove Units (AMC)<br><br>7. Bocas del Toro/Bastimentos	AMC	Bocas del Toro, Isla de Bastimento and San Blas. Stable conservation status, with a moderate level of threats and vulnerable in terms of its final conservation status.	-	65	0 - 200

Island/San Blas-Panama					
Pacific Mangrove Complexes and Mangrove Units<br><br>8. Pacific Mangrove Central America (PMCA)	PMCA	Pacific humid coast (Costa Rica and Panama). Vulnerable conservation status, with medium threat level and vulnerable final conservation status. Panama's Pacific dry coast. Relatively stable, moderate threat level and stable final conservation status.	-	850	0- 200
9. Pacific Mangrove South America (PMSA)	PMSA	Gulf of Panama (Panama and Colombia). Relatively stable, medium level of threats and relatively stable state of final conservation.	-	1,296	0 - 200
