# Supplementary figures and images for: Two Decades of Wildlife Pathogen Surveillance: Case Study of Choclo orthohantavirus and Its Wild Reservoir Oligoryzomys costaricensis
Source: Viruses. 2023 Jun 17;15(6):1390. doi: 10.3390/v15061390 (PMC10303383; doi:10.3390/v15061390)

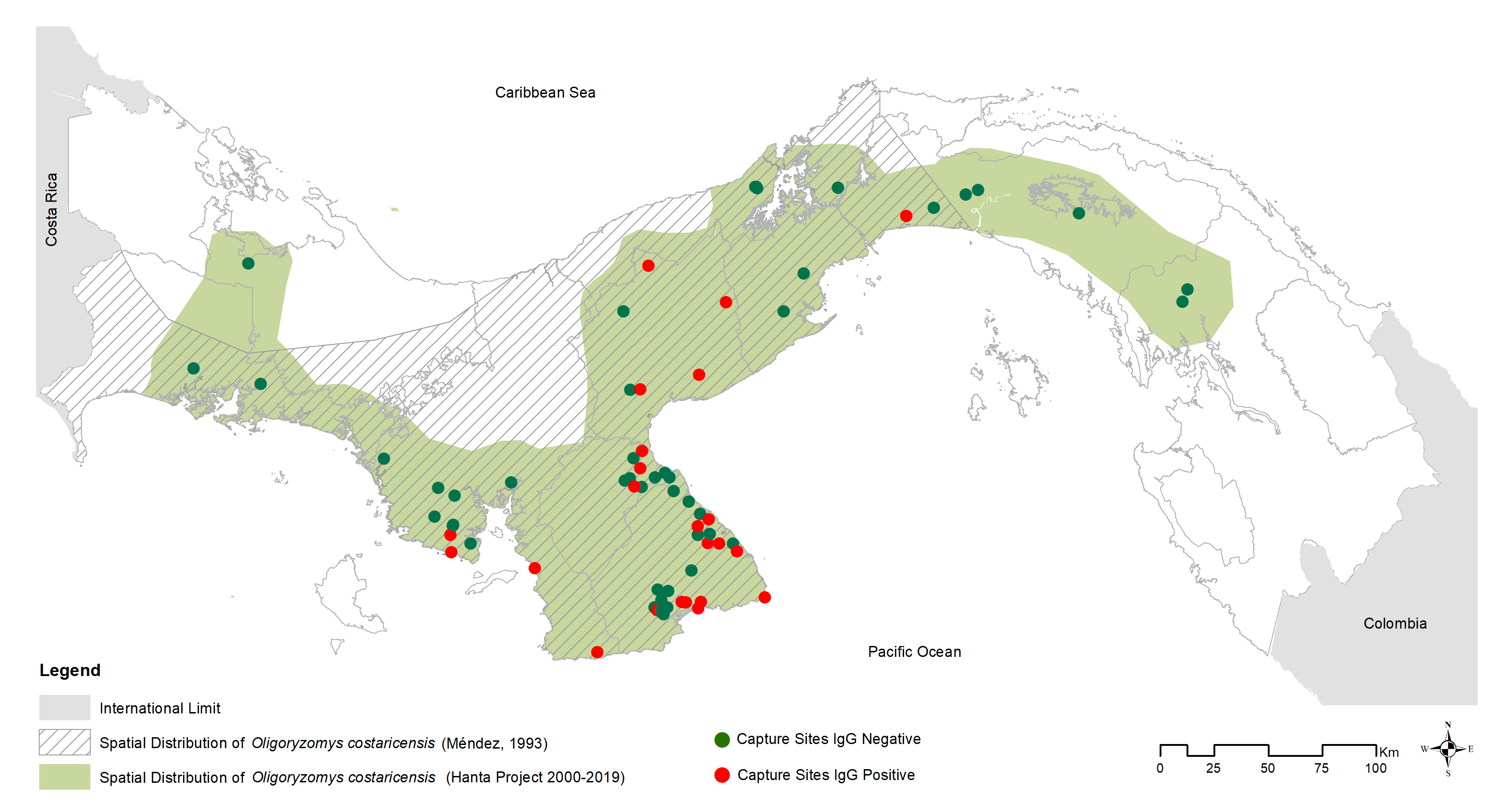

Supplement: Supplementary file 1 [file viruses-15-01390-s001.zip › Supplementary Figure S1. Distribución Espacial de O costaricensis histórico y actual 12062023.tif]
